# Editorial: The Psychology of Pseudoscience

**DOI:** 10.3389/fpsyg.2022.935645

**Published:** 2022-05-31

**Authors:** Stefaan Blancke, Taner Edis, Johan Braeckman, Sven Ove Hansson, Asheley R. Landrum, Andrew Shtulman

**Affiliations:** ^1^Department of Philosophy, Tilburg Center for Moral Philosophy, Epistemology and Philosophy of Science (TiLPS), Tilburg University, Tilburg, Netherlands; ^2^Department of Physics, Truman State University, Kirksville, MO, United States; ^3^Department of Philosophy and Moral Science, Ghent University, Ghent, Belgium; ^4^Department of Philosophy and History, Royal Institute of Technology, Stockholm, Sweden; ^5^Department of Advertising and Brand Strategy, College of Media & Communication, Texas Tech University, Lubbock, TX, United States; ^6^Department of Psychology, Occidental College, Los Angeles, CA, United States

**Keywords:** pseudoscience, irrationality, misbeliefs, cultural epidemiology, cognition and culture

If we want to understand how humans can produce scientific knowledge but are also vulnerable to a wide range of misbeliefs, then psychology is a good place to start. Psychologists have long been interested in questions concerning rationality and irrationality; in recent years they have increasingly turned their attention to phenomena such as science denialism, anti-science ideologies, and conspiracy theories. A “psychology of pseudoscience” (in a broad sense) seems to be the natural outcome of both naturalistic tendencies in the philosophy of science and an increased interest in pseudoscience within psychology. With this special collection, edited by a group of philosophers and psychologists, we wanted to take stock of these developments and contribute to this emerging field. Our motivation is not just to better understand pseudoscience but also to help impede its dissemination and mitigate its harmful effects.

A psychology of pseudoscience explores what makes people vulnerable to misbeliefs. In her paper “Conspiratorial Beliefs and Cognitive Styles: An Integrated Look on Analytic Thinking, Critical Thinking, and Scientific Reasoning in Relation to (Dis)trust in Conspiracy Theories,” Gjoneska investigates the relation between dispositions toward conspiracy beliefs and three cognitive styles: analytic thinking, critical thinking, and scientific reasoning. An extensive literature suggests that conspiracy theories might result from a lack of each of these cognitive styles. The article characterizes each of these styles and articulates their differences. Gjoneska concludes that research on these different cognitive styles has not been well integrated and proposes a new theoretical framework. She makes recommendations on how to effectively address conspiratorial thinking and hints at new possibilities for research.

We must ask not just about how individuals hold mistaken beliefs but how misbeliefs spread from one mind to the next. This propagation of misinformation is how pseudoscience becomes a cultural phenomenon. Cultural epidemiology can help us bridge the gap between the cognitive and the cultural. This theory directs attention to items of belief that and spread through chains of social transmission. To understand cultural phenomena, we then must explain why some items manage to become widespread and thus cultural, whereas others do not. Three contributions to this special collection rely on a cultural epidemiological framework to identify some of the factors that account for the popularity of pseudoscience. In “Counterintuitive pseudoscience propagates by exploiting the mind's communication evaluation mechanisms,” Mermelstein and German explain that at least some forms of pseudoscience, such as astrology and parapsychology, spread because they are to some extent counterintuitive. For instance, astrology goes against our intuition that stars cannot exert a causal effect from a distance. The authors complement an earlier suggestion that pseudoscience spreads because it is intuitively appealing. They discuss how counterintuitive pseudoscientific beliefs are represented as reflective beliefs and why people remember and transmit them. Remarkably, they note that such beliefs might be entertained only in a shallow way for use in particular contexts with little to no effect on people's behavior. Furthermore, they argue that people might come to accept counterintuitive pseudoscientific claims because they trust the source, are convinced by reasons offered, or are helped by these beliefs to reduce their stress and anxiety.

The notion that people accept misbeliefs based on trust is central to the paper by Fuhrer et al. with the title “Pseudo-expertise: A conceptual and theoretical analysis.” Pseudo-experts piggyback on the well-deserved reputation of proper experts to create the misleading impression that they are competent and trustworthy sources of information. As such, pseudo-experts not only help to spread misinformation, but they also undermine people's trust in real experts and pose “a threat to the very foundations of knowledge in liberal societies.” To deal with this problem effectively, however, we must understand pseudo-expertise and the dynamics underlying its cultural success. The authors therefore first provide a conceptual analysis of pseudo-expertise and distinguish it from other forms of non-expertise such as pseudoscience. They then discuss the emergence, spread, and fate of pseudo-expertise.

In the final paper of our collection, “Stakes of knowing the truth: A motivational perspective on the popularity of a controversial scientific theory,” Morisseau et al. use the example of hydroxychloroquine in fighting COVID to argue that people are not always motivated to find the truth. Instead, they adopt misbeliefs because of social and emotional concerns. This happens when people do not have a high stake in believing things that are not true. The authors suggest that people hold these beliefs only superficially, resulting mainly in expressions of belief that have social purposes. Nevertheless, it is important to address such misbeliefs because they impede effective responses to important social concerns. For that, we will have to restore trust in cultural and epistemic authorities rather than simply provide correct information.

The papers in this collection suggest that pseudoscience and other forms of misbelief will not go away quickly. They exploit our cognitive and communicative capacities and require much cognitive effort to overcome. However, the theoretical analyses presented here help explain why people adopt and spread pseudoscientific beliefs. Only when we understand these processes we can develop effective ways to counter them.

## Author Contributions

SB and TE wrote the first version of the manuscript. All authors contributed to the final version.

## Conflict of Interest

The authors declare that the research was conducted in the absence of any commercial or financial relationships that could be construed as a potential conflict of interest.

## Publisher's Note

All claims expressed in this article are solely those of the authors and do not necessarily represent those of their affiliated organizations, or those of the publisher, the editors and the reviewers. Any product that may be evaluated in this article, or claim that may be made by its manufacturer, is not guaranteed or endorsed by the publisher.

